# Is Phenylnitrene a Missing Link in the Formation of Polycyclic Aromatic Nitrogen Heterocycles?

**DOI:** 10.1002/anie.202503940

**Published:** 2025-06-17

**Authors:** Rahel Arns, Rory McClish, Patrick Hemberger, Andras Bodi, Jordy Bouwman, Tina Kasper, Domenik Schleier

**Affiliations:** ^1^ Lehrstuhl Technische Thermodynamik Fakultät für Maschinenbau Universität Paderborn Warburger Str. 100 33098 Paderborn Germany; ^2^ Department of Chemistry University of Colorado Boulder Colorado 80309 USA; ^3^ Laboratory for Atmospheric and Space Physics University of Colorado Boulder Colorado 80303 USA; ^4^ Laboratory for Synchrotron Radiation and Femtochemistry Paul Scherrer Institute (PSI) Villigen 5232 Switzerland; ^5^ Institute for Modeling Plasma Atmospheres and Cosmic Dust (IMPACT) NASA/SSERVI Boulder Colorado 80309 USA; ^6^ Institut für Physik und Astronomie Technische Universität Berlin Hardenbergstr. 36 10623 Berlin Germany

**Keywords:** Astrochemistry, Nitrenes, Polycyclic aromatic hydrocarbons, Radical chemistry, Reaction dynamics

## Abstract

The incorporation of heteroatoms into the framework of polycyclic aromatic hydrocarbons (PAHs), in particular of nitrogen to yield polycyclic aromatic nitrogen heterocycles (PANHs), has been proposed for both astronomical and combustion environments, but no suitable precursors and pathways have been found. Analogous pathways to PAH formation are kinetically or energetically inhibited in the presence of a nitrogen heteroatom. We report on the reaction of phenylnitrene (^3^PhN, *c*‐C_6_H_5_N) with resonance‐stabilized propargyl radicals (C_3_H_3_) and find that the association reaction bifurcates depending on the orientation of the attacking propargyl radical and yields multiple isomeric products. Among them, we identify the condensed‐ring quinoline and conclude that nitrenes are viable candidates to drive the formation of PANHs.

## Graphical abstract

A new formation pathway for polycyclic aromatic nitrogen heterocycles (PANHs) has been found. The reaction of phenylnitrene and the propargyl radical leads to quinoline, proving that nitrenes are suitable precursors for nitrogen incorporation mechanisms. We use double imaging photoelectron photoion coincidence (PEPICO) spectroscopy to identify the products and quantum chemistry to explore the mechanism in detail.

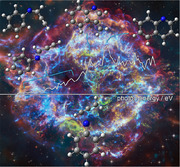

Polycyclic aromatic hydrocarbons (PAHs) are ubiquitous in both terrestrial^[^ ^1^ ^]^ and extraterrestrial environments.^[^ ^2^, ^3^, ^4^ ^]^ Their formation mechanisms have been a research focus for decades in both astrochemistry and combustion to explain their abundance and to reduce their anthropogenic emissions.^[^ ^5^, ^6^ ^]^ Multiple mechanisms were discovered that yield small PAHs^[^ ^7^, ^8^, ^9^, ^10^ ^]^ and explain their propensity to grow into larger aromatic structures, which is enabled and driven by resonance‐stabilized radicals (RSR).^[^ ^11^ ^]^ These mechanisms are active in similar conditions, such as at high temperatures (>1000 K) and in fuel‐rich environments, which facilitates the incomplete oxidation of the starting materials. Consequently, extraterrestrial PAHs are believed to originate in the outflow of carbon‐rich stars under similar high‐temperature conditions.^[^ ^12^, ^13^ ^]^ However, astronomers found that heteroatoms, in particular nitrogen, may be locked up in aromatic molecules, which could play a vital role in the chemistry of space.^[^ ^14^, ^15^, ^16^ ^]^ Yet, the mechanism and conditions by which polycyclic aromatic nitrogen heterocycles (PANHs) may be produced are not known. This is further exacerbated by a lack of PANH detection in some nitrogen–rich model environments, such as ammonia/hydrocarbon flames,^[^ ^17^, ^18^, ^19^ ^]^ which questions if PANHs can be formed under the same conditions as PAHs. On the one hand, low‐temperature (<20 K) formation routes based on ionic nitrogen species^[^ ^16^, ^20^ ^]^ and neutral nitrogen‐bearing hydrocarbon radicals^[^ ^21^, ^22^, ^23^ ^]^ have been suggested as an alternative source for PANHs. However, there is hardly any evidence that the proposed radicals are abundant in (extra)terrestrial environments. On the other hand, phenylnitrene (^3^PhN, **1**, Scheme 1), the smallest aromatic nitrene (R–N:), was identified in pyrrole and pyridine flames,^[^ ^24^, ^25^, ^26^ ^]^ as well as in processed benzene‐doped N_2_ ices,^[^ ^27^ ^]^ corresponding to two model environments for circumstellar and interstellar conditions. Moreover, nitrenes have become useful tools in organic chemistry to form new C─N bonds,^[^ ^28^, ^29^, ^30^, ^31^, ^32^ ^]^ and their reactions with RSR may be plausible sources for PANHs under high‐ and low‐temperature conditions. One key reactant for PAH formation in combustion^[^ ^33^ ^]^ and astrochemistry,^[^ ^34^ ^]^ is the resonance‐stabilized propargyl radical (C_3_H_3_, **2**, Scheme 1), whose presence has been established in various objects.^[^ ^35^, ^36^ ^]^

**Scheme 1:**
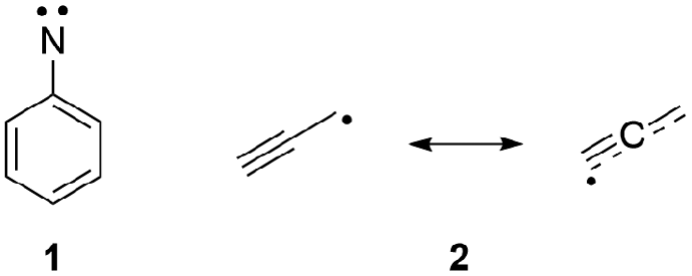
Chemical structure of triplet phenylnitrene (^3^PhN, **1**) and the resonance structures of propargyl (C_3_H_3_, **2**).

The lack of knowledge on bimolecular nitrene chemistry combined with their unique properties and potential to form PANHs motivated us to investigate the reaction of triplet phenylnitrene with the propargyl radical in a pyrolysis microreactor, using double imaging photoelectron photoion coincidence (i^2^PEPICO) spectroscopy.

To provide an overview of the pyrolysis products and side products formed when pyrolyzing the isolated nitrene and propargyl precursors, Figure 1 displays mass spectra recorded at a photon energy of 9.00 eV. In the upper trace, the pyrolysis of phenyl azide is shown, which has been the subject of a dedicated investigation using the same instrument.^[^ ^37^ ^]^ The complete conversion of the precursor is achieved at ≈520 °C, and besides ^3^PhN observed at *m*/*z* 91, signals arise at *m*/*z* 93, as well as 143, 154, 167–169, and 182. The first is identified as aniline,^[^ ^37^ ^]^ and the last corresponds to azobenzene, the dimer of ^3^PhN.^[^ ^38^ ^]^ The masses between the two arise primarily from the pyrolysis of azobenzene and have been identified in a previous study.^[^ ^39^ ^]^ A detailed assignment of products is provided in Figure S1. In the middle trace, propargyl iodide was pyrolyzed, resulting in signals at *m*/*z* 39, 78, 116, and 204, corresponding to the propargyl radical (Figure S2), C_6_H_6_ isomers,^[^ ^34^ ^]^ indene, and iodobenzene (Figure S3), respectively. These observations are in line with previous investigations of the propargyl self‐reaction under similar conditions.^[^ ^34^, ^40^ ^]^ In the co‐flow experiments displayed in the bottom trace of Figure 1, the mass spectrum is almost identical to the sum of the individual precursor spectra. However, a small peak at *m*/*z* 129 emerges (highlighted in the red trace), which becomes more prominent at higher temperatures, best seen in the top trace of Figure S5.

**Figure 1:**
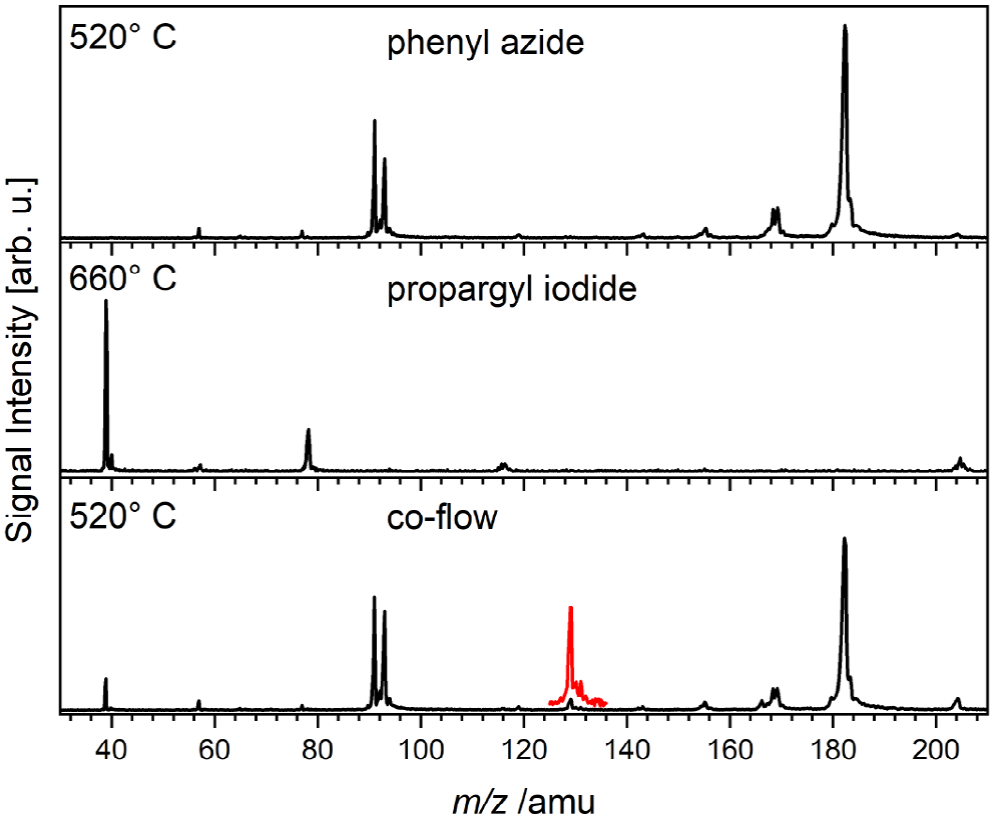
Pyrolysis mass spectra of phenyl azide (PhN_3_, top), propargyl iodide (C_3_H_3_I, middle), and a co‐flow of both (bottom) recorded at a photon energy of 9.00 eV. The newly formed *m*/*z* 129 peak in the bottom trace is highlighted in red (× 10) for better visibility.

To suppress the isomerization of PhN to cyanocyclopentadiene (Cp–CN),^[^ ^37^ ^]^ we chose as gentle pyrolysis conditions as possible to still observe bimolecular chemistry involving our species of interest. A detailed description of our approach is given in Chapter S5. In summary, phenyl azide decomposes already at 450 °C, while propargyl iodide decomposition sets in at 520 °C. As such, 520 °C was determined to be the lowest possible temperature to observe bimolecular chemistry. Fortunately, the closed‐shell nature of aniline and Cp–CN makes their association reactions with propargyl radicals unlikely. To verify this hypothesis, we calculated the initial association reaction steps between aniline as well as the three Cp–CN isomers and propargyl (Figures S8–S10). For aniline, we find barriers between +49 and +78 kJ mol^−1^ for propargyl attaching to the phenyl ring, and a barrier of +188 kJ mol^−1^ for the reaction with the amino group (Figures S11 and S12). It must also be noted that the formed intermediates are either highly endothermic (+143 kJ mol^−1^) or approximately isoenergetic to the isolated reactants. For Cp–CN, all isomers exhibit energy barriers between +64 and +95 kJ mol^−1^ and the subsequently formed intermediates are endothermic, underlining their low reactivity toward propargyl.

Next, we determined the isomeric composition of *m/z* 129 by recording a mass‐selected threshold photoelectron spectrum (ms‐TPES), displayed in the top trace of Figure 2. The spectrum shows a slow rise starting at around 8.2 eV with superimposed features and reaches its maximum at 8.62 eV. At higher photon energies, the signal remains at a high level without a clear vibronic structure and declines only slightly. The structure of the spectrum suggests that multiple isomeric species with adiabatic ionization energies (IE_*ad*_) between 8.1 and 8.7 eV are present.

**Figure 2:**
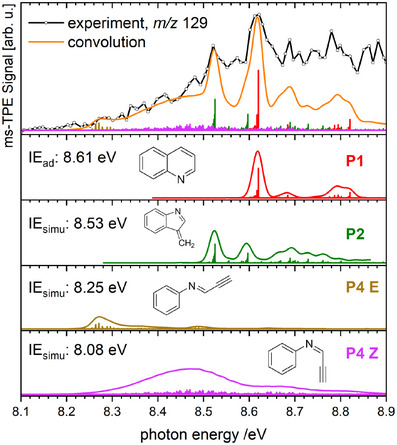
ms‐TPES of mass channel 129 in the co‐flow experiment at 520 °C (top). Only thermalized ions were selected to construct the ms‐TPES. Individual simulations of potential isomeric product species (bottom). Calculated IE_*ad*_ at the CBS‐QB3 level (Table 1) were shifted within the error limit to reproduce the experiment best.

To estimate which molecules contribute to the spectrum, we calculated the IE_*ad*_ of 18 different C_9_H_7_N isomers listed in Table S1.^[^ ^41^ ^]^ Of those, nine can be excluded due to their IE_*ad*_ exceeding 8.90 eV. These also have the highest formation energy and can only form through major rearrangements that include multiple C–C bond scissions. Further, two isomers have calculated IE_*ad*_ below 8.1 eV and can be ruled out based on the absent *m*/*z* 129 peak at this energy. The remaining seven with an IE_*ad*_ in the investigated region are listed in Table 1. Based on Franck–Condon (FC) simulations displayed in Figure S13, the broad featureless rise can be best attributed to the substituted benzene product **P4 Z**, while the vibronic features on top could originate from either of the condensed ring products **P1**, **P2**, **P3** and **P5**. For **P6**, the FC‐simulation predicts an intense peak near the origin transition at 8.11 eV. Yet, the ms‐TPE signal remains low and only starts to rise slowly above 8.2 eV, indicating negligible production. We can describe the spectrum reasonably well using a mixture of the remaining isomers **P1**, **P2**, **P3**, **P4 E**, **P4 Z**, and **P5** illustrated in Figure S14. Even though the simulation agrees qualitatively, some discrepancies can be observed at 8.55 eV and above 8.65 eV. The agreement between FC simulations and experiments commonly deteriorates at higher energies, which is either caused by increasing anharmonicities due to an insufficient description of the cationic potential wells at large vibronic quantum numbers or due to the presence of electronically excited cation states. We also tried to limit the amount of contributors by omitting **P3**, **P4 E**, **P4 Z**, and **P5**, as depicted in Figure S15. Although **P1** and **P2** describe most spectral features, they do not explain the rise between 8.2 and 8.5 eV, which requires **P4 Z**, especially since only thermalized ions were selected, effectively eliminating potential hot‐band contributions.^[^ ^43^ ^]^ To further prove quinoline (**P1**) formation, Figure S16 compares the experimental spectrum to the previously recorded ms‐TPES of quinoline,^[^ ^42^ ^]^ and its FC‐simulation used in Figure 2. Although our experiment exhibits a slightly broader origin band and its maximum is shifted by ≈10 meV to lower energies, the vibronic feature at 8.79 eV further corroborates the assignment. The presence of three isomers **P3**, **P4 E**, and **P5** is questionable based on the ms‐TPES and the FC‐simulations alone. **P3** is only indicated through a small peak at 8.48 eV, while the remaining vibrational features may be convoluted with the resonances of **P1** and **P2**. Similarly, **P4 E** and **P5** cannot be unambiguously identified even though their inclusion in the simulation improves the agreement with the experiment slightly. As such, neither reference spectra nor FC‐simulations allow for a clear identification or exclusion of these isomers based on the experimental data.

**Table 1:** Potential reactions products of the ^3^PhN + propargyl reaction with IE_*ad*_ between 8.1 and 8.9 eV. Calculated IE_*ad*_ are based on CBS‐QB3 calculations.

Molecule	Label	IE_*ad*, *calc*_/eV	IE_*ad*, *exp*_/eV
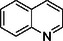	**P1**	8.68	8.61^[^ ^42^ ^]^
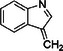	**P2**	8.57	−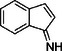	**P3**	8.48	−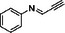	**P4 E**	8.19	−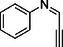	**P4 Z**	8.14	−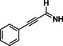	**P5**	8.65	−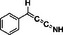	**P6**	8.11	−

Spectral assignment goes hand in hand with understanding of the underlying chemistry, which is why we explored the potential energy surface (PES) of the main reaction. A summary of the lowest‐energy pathways is shown in Figure 3 and a detailed description of each route is given in Chapter S8. The propargyl radical is resonantly stabilized and exhibits spin density on both terminal carbon atoms. This enables its attack on phenylnitrene with either its CH or CH_2_ site.^[^ ^44^, ^45^ ^]^ In both cases, the association reaction between the two reactants is barrierless. Thus, depending on the impact parameter for the two entrance channels, the CH and CH_2_ reaction channels may yield two sets of final products, which do not compete with each other. We will start our PES exploration with the CH reaction and explore the CH_2_ reaction afterwards.

**Figure 3:**
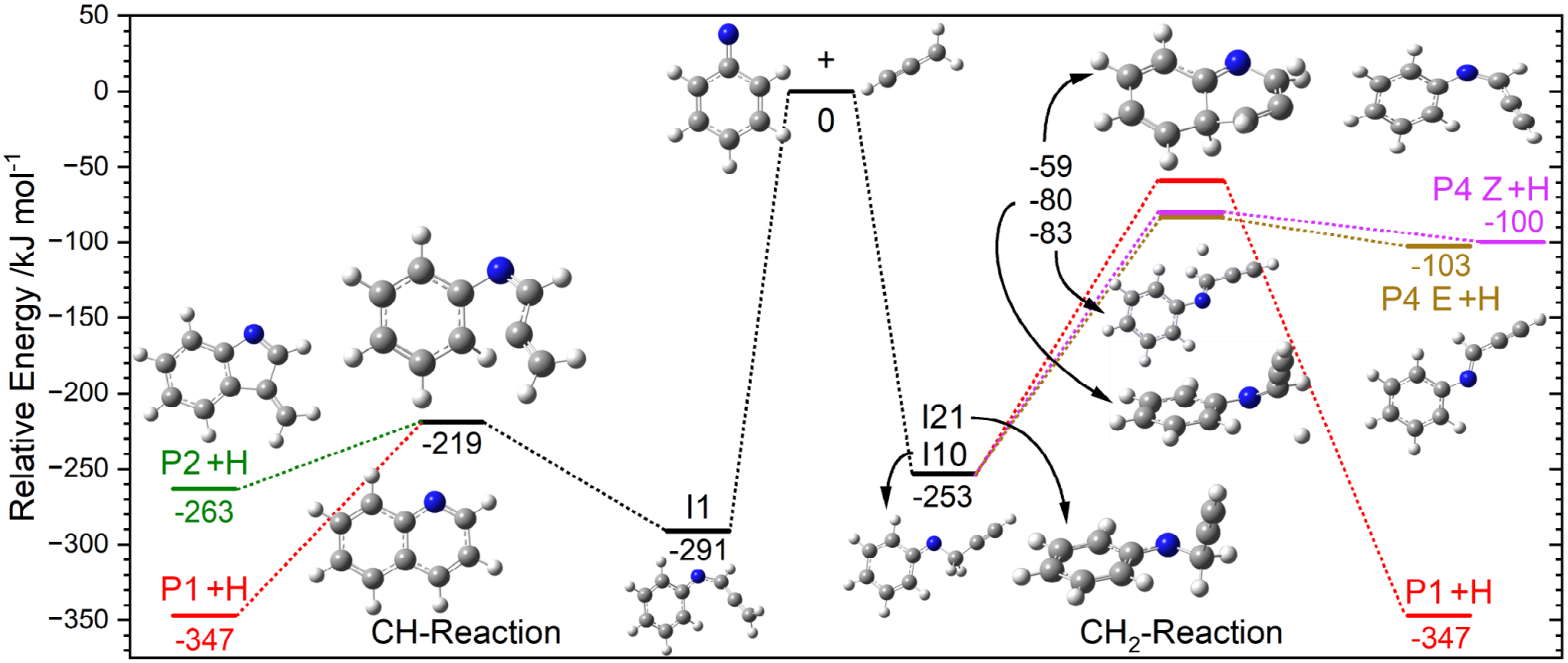
Overview of the product formation pathways in the phenylnitrene and propargyl association reaction at the CBS‐QB3 level. Only the highest‐energy, rate‐determining transition state is given for each pathway. Color coding of the products is according to Figure 2. Propargyl can attack phenylnitrene with the CH–site (left) or with its CH_2_–site (right). The E–configuration for **I1** is omitted for simplicity. A detailed description of the reaction pathways can be found in the Supporting Information.

The lowest energy pathway (Figure S18) leads over a submerged transition state at −219 kJ mol^−1^, resulting in a barrier of +72 kJ mol^−1^ with respect to **I1** and forms a five‐membered ring containing a terminal methylene (CH_2_) group. The mechanism branches into two pathways after ring closure (**I2**, Figure S19), one to **P1** and one to **P2**. The barriers for **P1** formation are lower, and the CH_2_–group can insert into the five‐membered ring by a mechanism akin to fulvene–benzene isomerization.^[^ ^33^ ^]^ Yet, the barrier for hydrogen atom elimination from **I2** to **P2** is only 25 kJ mol^−1^ higher, so that the two channels are expected to compete according to the number of states of their rate‐determining transition states. Based on the ms‐TPES, rates for **P1** and **P2** are competitive, and both are formed through these pathways. Three more pathways with significantly higher rate‐limiting transition states leading to **P1** (Figures S20–S22) are not expected to be able to compete statistically. Similarly, the channels to the ring‐open products **P4 E**, **P4 Z**, and **P7** (Figure S23) exhibit barriers that are too high to be competitive with **P1**/**P2** formation. The same is true for the imine product **P3** (Figure S24). Hence, the CH reaction explains the appearance of the condensed products **P1** and **P2**, but **P3**, **P4 E**, **P4 Z**, and **P5** are unlikely to be formed.

In contrast to the CH reaction, the CH_2_ reaction will not form a five‐membered ring, as the required cyclization is associated with significantly higher‐energy transition states between −21 kJ mol^−1^ and +56 kJ mol^−1^ (Figures S25 and S26). These correspond to intramolecular hydrogen shifts to saturate the terminal CH and enable condensed ring formation. Consequently, we do not expect **P1** and **P2** production in the same way as for the CH_2_ reaction. On the contrary, the energetically most favorable pathways are to the ring‐open products **P4 E** and **P4 Z**, exhibiting transition states at −80 and −83 kJ mol^−1^ (Figure S27 and S28). These final products are expected to dominate, although cyclization and condensed ring formation may still be possible, bypassing the five‐membered ring intermediate over a −59 kJ mol^−1^ rate‐determining transition state to yield **P1** as a minor product of the CH_2_ reaction (Figure S29).

The PES exploration highlights that the orientation of the propargyl radical as it attacks phenylnitrene is crucial for determining the outcome of the reaction. The CH reaction leads to the formation of **P1** and **P2**, while the ring‐open species **P4 Z** and **P4 E** are formed through the CH_2_ reaction. We could not find competitive routes to **P3** (Figure S24) and **P5** computationally. The relatively large reorganization required for both and the high energy of **P5** (−130 kJ mol^−1^) mean that they are unlikely to be formed as a result of the reaction between PhN and propargyl. However, we cannot exclude their presence in the experiment as a result of secondary processes, which are not covered by our PES and do not affect **P1** formation. A detailed discussion on the presence of **P3** and **P5** is given in Section S8.1.3.

The proof for the possibility of nitrogen heteroatom incorporation in the PAH growth mechanism under high temperature conditions has long been missing in astro‐ and combustion chemistry. Although the pyridyl radical has been suggested to be important in such environments,^[^ ^21^, ^22^ ^]^ it has not been detected there so far. Nitrenes can fill this gap, and their detection in flames and processed ices corroborates their importance under such conditions.^[^ ^24^, ^25^, ^26^, ^27^ ^]^ Nitrenes are generally more stable than their carbene analogues and nitrogen‐containing carbenes isomerize easily to the respective nitrenes.^[^ ^46^ ^]^ The presence of carbenes has been established multiple times in astrochemistry and hints for phenylcarbene have been found in toluene flames,^[^ ^47^ ^]^ making the presence of nitrenes, such as ^3^PhN, plausible. The barrierless reaction between ^3^PhN and propargyl, followed by submerged transition states and hydrogen atom elimination to stabilize the final products, also makes it a possible source of PANHs in the interstellar medium (ISM). So far, only HACA or HAVA mechanisms are known to lead to PANHs, such as quinoline.^[^ ^21^, ^22^ ^]^ However, their precondition for molecular growth is that the nitrogen atom must be already incorporated in the aromatic framework. Our nitrene mechanism accounts for precisely this step, the incorporation of the nitrogen atom into the aromatic framework. Since the detection of PANHs in the ISM is hampered by their weak rotational transitions^[^ ^48^, ^49^ ^]^ we suggest searching for PANH precursors instead. In fact, a similar strategy is currently being followed to infer the presence of larger PAHs through their nitrile derivatives.^[^ ^50^ ^]^ However, before such efforts can succeed, fundamental data, such as gas phase rotational spectra, need to be obtained for nitrenes to guide future observations.

In summary, we have investigated the role nitrenes play in PANH formation. For this, we generated triplet phenylnitrene and propargyl radicals in a pyrolysis microreactor and identified a product peak at *m*/*z* 129, aligning with the formation of C_9_H_7_N species. To determine the isomeric composition, we recorded the ms‐TPES, which revealed a complex mixture of several isomeric products containing monocyclic and bicyclic aromatic species. Investigation of the PES shows that, depending on the orientation of the propargyl radical, different products are expected in its reaction with phenylnitrene. Barrierless cyclization is the lowest energy pathway for the reaction of the CH‐terminal propargyl radical, whereas in the CH_2_‐reaction, linear side‐chains are energetically favorable. As such, nitrenes can explain the occurrence of PANHs in high‐temperature environments akin to the formation of their carbonaceous congeners. PAHs and PANHs can thus have similar origins, and both should be considered in PAH models. Lastly, we believe that ^3^PhN is a promising target for astronomical observations as well as an important intermediate in nitrogen‐rich flames.

## Conflict of Interests

The authors declare no conflict of interest.

## Supporting information

Supporting Information

## Data Availability

The data that support the findings of this study are available in the Supporting Information of this article.
