# Supplementary material for: Is Phenylnitrene a Missing Link in the Formation of Polycyclic Aromatic Nitrogen Heterocycles?
Source: Angew Chem Int Ed Engl. 2025 Jun 17;64(31):e202503940. doi: 10.1002/anie.202503940 (PMC12304799; doi:10.1002/anie.202503940)
Supplement: Supplementary file 1 — Supporting Information [file ANIE-64-e202503940-s001.pdf]

Electronic Supplementary Information to Is Phenylnitrene a missing link in the formation of Polycyclic Aromatic Nitrogen Heterocycles?

Rahel Arns,[a] Rory McClish,[b,c] Patrick Hemberger,[d] Andras Bodi,[d] Jordy Bouwman*,[b,c,e] Tina Kasper,[a] and Domenik Schleier*[f]

[a] R. Arns, Prof. Dr. T. Kasper
Lehrstuhl Technische Thermodynamik
Fakultät für Maschinenbau
Universität Paderborn
Warburger Str. 100, 33098 Paderborn, Germany

[b] R. Mcclish, Prof. Dr. J. Bouwman*
Department of Chemistry
University of Colorado
Boulder, CO 80309, USA
E-mail: jordy.bouwman@colorado.edu

[c] R. Mcclish, Prof. Dr. J. Bouwman
Laboratory for Atmospheric and Space Physics
University of Colorado
Boulder, CO 80303, USA

[d] Dr. P. Hemberger, Dr. A. Bodi
Laboratory for Synchrotron Radiation and Femtochemistry
Paul Scherrer Insitute (PSI)
5232 Villigen, Switzerland

[e] Prof. Dr. J. Bouwman
Institute for Modeling Plasma, Atmospheres and Cosmic Dust (IMPACT), NASA/SSERVI
Boulder, CO 80309, USA

[f] Dr. D. Schleier
Institut für Optik und Atomare Physik
Technische Universität Berlin
Hardenbergstr. 36, 10623 Berlin, Germany.
E-mail: schleier@physik.tu-berlin.de

This Electronic Supplementary Information contains further information to the main manuscript.

Contents

S1 Experimental **S3**

S2 Computational **S5**

S3 Phenylazide Pyrolysis **S6**

S4 Propargyl Iodide Pyrolysis **S7**

S5 Co-Flow Pyrolysis **S9**

S6 Potential secondary chemistry **S14**

S7 Isomers of C_9H_7N (m/z 129) **S17**

S8 Potential Energy Surface Calculations **S24**

S8.1 CH Reactions S25

S8.1.1 Formation of P1 and P2 . . . . S25

S8.1.2 Formation of P4E, P4Z and P7 . . . . S30

S8.1.3 On the presence of P3 and P5 . . . . S32

S8.2 CH_2 Reactions S34

S1 Experimental

The experiments have been performed using the double imaging CRF-PEPICO instrument at the Vacuum Ultraviolet (VUV) beamline of the Swiss Light Source, Paul Scherrer Institute. A detailed description of the beamline[1, 2] and of the CRF-PEPICO instrument can be found elsewhere,[3] and the apparatus will only be described briefly here.

The precursor propargyl iodide was synthesized using a Finkelstein reaction from propargyl bromide and sodium iodide, described in detail in the literature.[4] Phenyl azide was synthesized according to literature procedures.[5] The precursors were placed in separate sample containers, cooled to 4 °C, and shielded from ambient light by wrapping the container in aluminum foil. Each container was connected to a separate argon bottle and a third argon line was used to set the total flow rate and control the dilution. The seeded precursors were introduced into the pyrolysis tube, a SiC tube with an inner diameter of 1 mm, where they were converted to the respective reactive species, phenylnitrene and propargyl radicals. Pyrolysis temperature readings are accurate within ±100 K regarding the reactor wall temperature. The gas flow expanded into vacuum and the ensuing molecular beam passed a skimmer as it entered the experimental chamber where it crossed the synchrotron radiation.

Synchrotron radiation was provided by a bending magnet, collimated, and diffracted using a plane grating (150 grooves/mm) resulting in an energy resolution of $E/\Delta E > 3000$. Higher harmonics were suppressed by a rare gas filter ($p_{\text{total}} = 10$ mbar over an optical length of 10 cm) operating with an Ar/Kr/Ne mixture. The photon energy was calibrated on the 11 s′–14 s′ autoionization resonances of Ar in the first and second order of the grating. The photon energy was scanned between 8.10 and 8.90 eV with 10 meV step size. Photoelectrons and -ions were extracted in a continuous 215 V cm^{-1} field. Threshold electrons

were collected with a resolution of $<5\,\mathrm{meV}$ and contributions of hot electrons were subtracted following the procedure reported by Sztaray and Baer.[6] To minimize thermal broadening and hot bands, the spectrum was constructed by selecting the thermalized ions in the ion image. This procedure has been documented to increase the isomer-specificity and the ms-TPES resolving power in pyrolysis studies.[7, 8]

S2 Computational

Geometry optimizations, followed by calculations of harmonic frequencies for neutral intermediates and transition states as well as cations, were performed on the B3LYP/6-311G(2d,d,p) level using the CBS-QB3 composite approach in the Gaussian 16 program package. The validity of the transition states was verified by internal reaction coordinate (IRC) scans. Ionization energies (IEs) were determined by subtracting the zero-point corrected energy of the neutral from that of the cation, leading to IE values that are usually accurate within ± 0.1 eV.[9] The threshold photoelectron spectrum was simulated in the harmonic approximation using ezSpectrum.[10] The FC-factors were calculated in the double harmonic approximation and the stick spectrum convoluted with a Gaussian with a full-width half maximum (FWHM) of 25 meV, to account for the rotational envelope at room temperature. To elucidate the species composition of m/z 129, isomers with IE_{ad} above 8.9 eV or below 8.1 eV were excluded, leaving six potential contributing isomers. After simulating their photoelectron spectra, the number of potential contributors was constrained by using the potential energy surface (PES) of the PhN + propargyl reaction as a guide, predicting the presence of P1, P2, P4E, and P4Z. Products P5 and P3 are not expected to be formed based on the PES calculations, however, we do not exclude their presence, e.g. through H-assisted secondary chemistry in the microreactor (see section S8.1.3). Subsequently, the individual simulations were placed at their calculated, or experimental IE_{ad} with their intensity and, in particular for P4E and P4Z, their IE_{ad} adjusted to reproduce the experimental spectrum. The final simulation of the experimental spectrum is, thus, a linear combination of the individual FC simulations.

S3 Phenylazide Pyrolysis

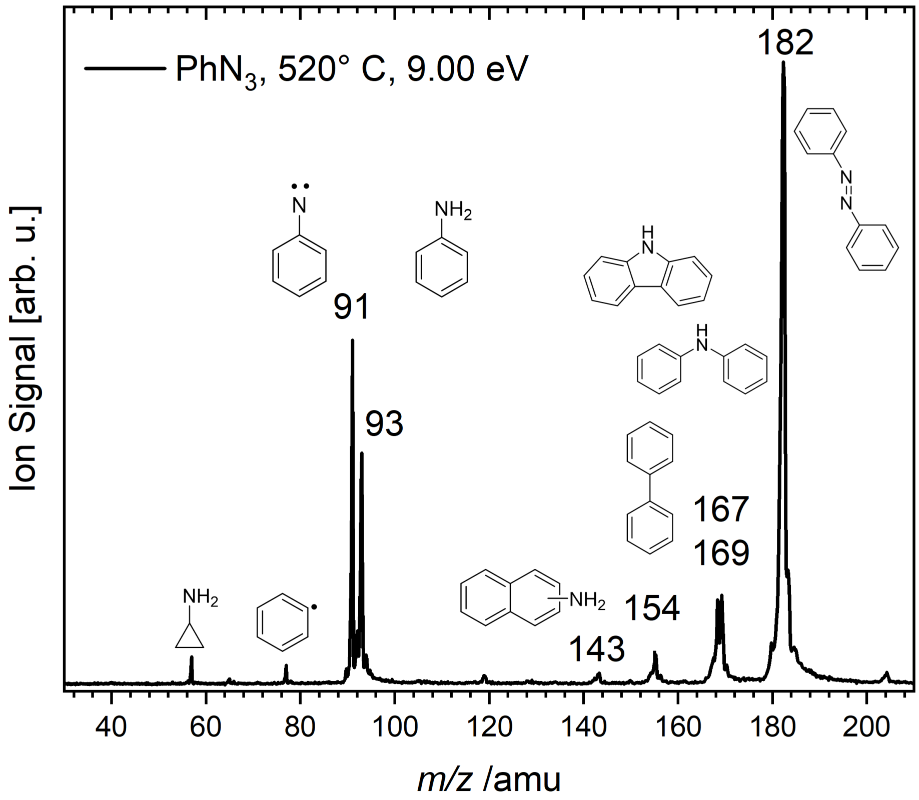

Figure S1: Pyrolysis mass spectrum of phenyl azide at $h\nu = 9.00\,\mathrm{eV}$ with the species assigned to the mass peaks. Residual cyclopropylamine (m/z 57) is present in the experimental chamber due to impurities from a previous experiment. The peaks agree with previous measurements and identifications under similar conditions.[11]. The small signal at m/z 119 corresponds to unpyrolyzed phenyl azide.

S4 Propargyl Iodide Pyrolysis

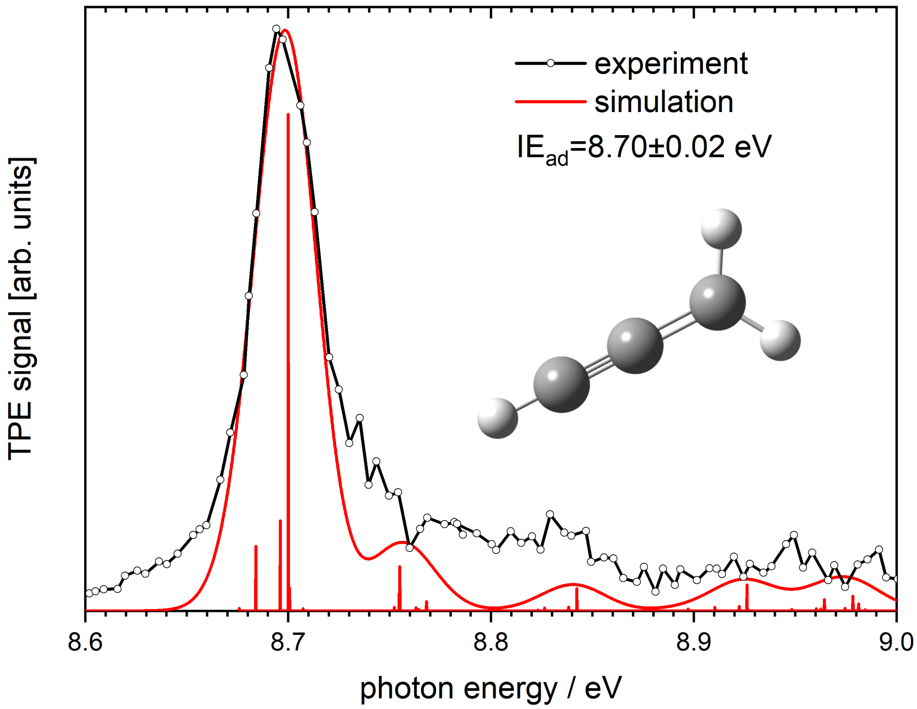

Figure S2: ms-TPES of mass channel 39 in pure propargyl iodide/Ar flow at 660 °C and simulation of the photoelectron spectrum of propargyl.

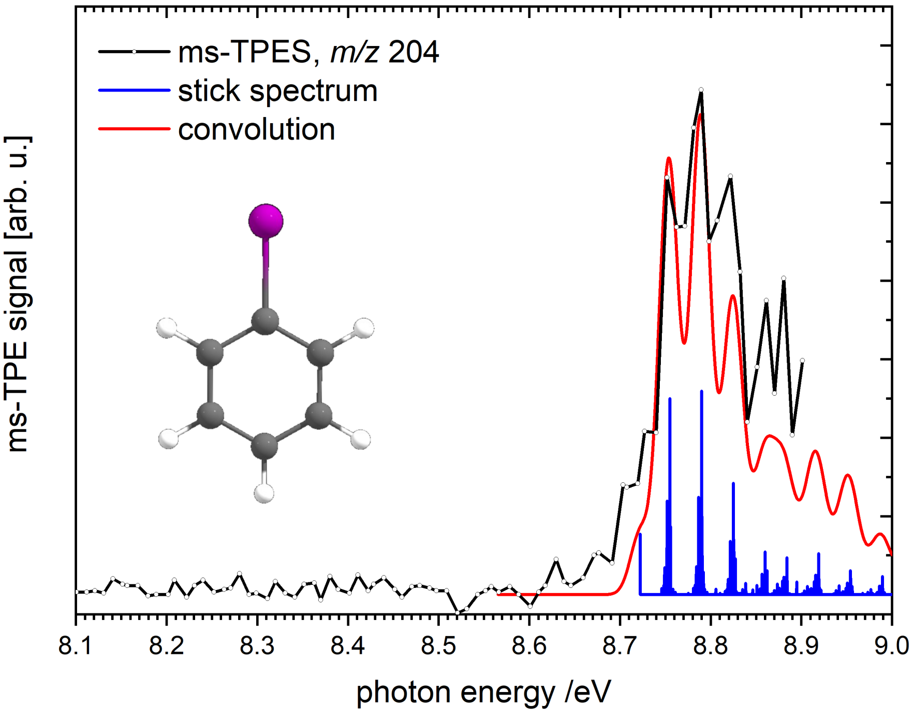

Figure S3: ms-TPES of mass channel 204 in pure propargyl iodide/Ar flow at 660 °C and FC-simulation of the iodobenzene photoelectron spectrum. The adiabatic ionization energy (IE_{ad}) of iodobenzene has been determined to be 8.754 eV [12] and the origin of the simulation was set to 8.755 eV.

S5 Co-Flow Pyrolysis

Temperature optimization of the co-flow experiments was necessary to minimize unwanted phenylnitrene isomerization. Mendez-Vega *et al.* investigated the decomposition of phenyl azide as a function of temperature and found partial rearrangements of PhN to cyanocyclopentadiene isomers (Cp–CN) above 500 °C, according to Fig. S4.[13] The main decomposition channel for phenyl azide is N_2 loss, leading to singlet phenylnitrene (^{1}PhN), which can either undergo isomerization to Cp–CN or intersystem crossing (ISC) to ^{3}PhN. The ISC is El-Sayed forbidden, thus it is slow and only efficient at lower temperatures, while rearrangements become dominant at higher temperatures.[14, 15] Soto *et al.* also proposed a concerted ISC-N_2-loss mechanism that produces ^{3}PhN directly, circumventing ^{1}PhN, but its influence is likely limited.[15] The two identified isomers (^{3}PhN and Cp–CN) can be distinguished by their IE_{ad}. While ^{3}PhN ionizes at 8.28 eV (Fig.S7), the Cp–CN isomers start to ionize at 9.14 eV.

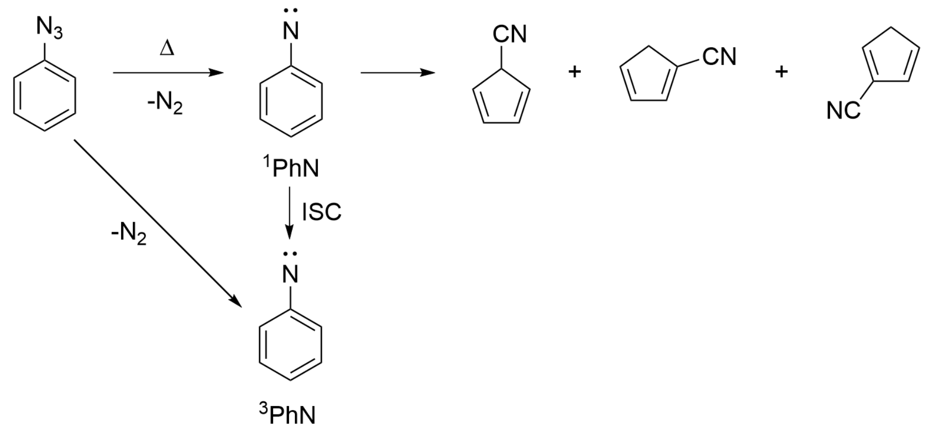

Figure S4: Decomposition of phenyl azide during flash vacuum pyrolysis.

To follow the undisturbed ^{3}PhN signal, we recorded temperature-dependent mass spectra at 9.00 eV displayed in Fig. S5. At 450 °C, phenylazide (m/z 119) is already partially decomposed as seen by the m/z 91 signal originating

from ^{3}PhN, whereas propargyl iodide (m/z 166) cannot be ionized at this photon energy and is presumed to be still intact since no m/z 39 signal is detected. When the temperature is raised to 520 °C, propargyl radicals become visible and their signal becomes even stronger at 590 °C. Although the propargyl radical signal is most intense at 590 °C, the m/z 91 signal is low at this temperature due to rearrangements. To confirm the onset of propargyl iodide pyrolysis at 520 °C, as well as the increased isomerization of PhN at 590 °C, we also recorded temperature-dependent mass spectra at 9.50 eV, displayed in Fig. S6. At 450 °C, an unaltered propargyl iodide signal in mass channel 166 is visible, confirming the assumptions from the lower photon energy measurements. At the same time, the signal of m/z 91 increases significantly at 590 °C, which is due to the additional detection of Cp–CN. The intermediate temperature of 520 °C was consequently chosen as a compromise between propargyl radical generation and suppressing ^{3}PhN rearrangement, although we cannot exclude partial isomerization of ^{3}PhN in the co-flow measurements. We further determined the identity of the m/z 91 signal by recording photoion mass-selected TPE spectra at two relevant temperatures (520 and 590 °C), which show that ^{3}PhN is present under both conditions, albeit its signal is significantly lower at higher temperatures, as shown in Figure S7.

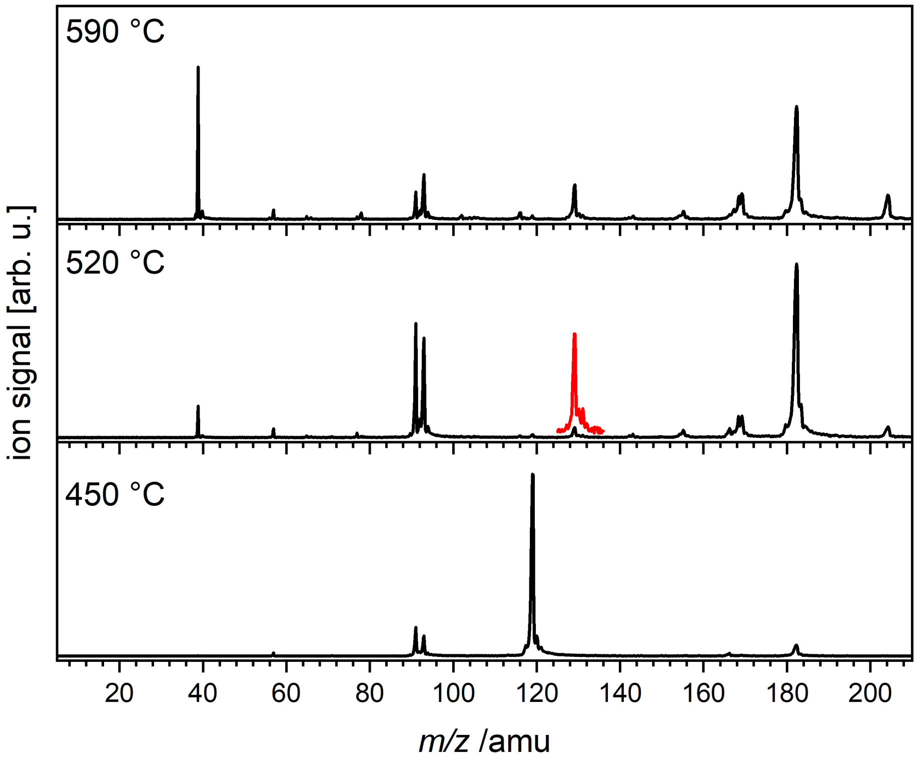

Figure S5: Temperature-dependent mass spectra of a co-flow of phenyl azide and propargyl iodide at $h\nu = 9.00\,\mathrm{eV}$. The newly formed m/z 129 peak in the bottom trace is highlighted in red ($\times 10$) for better visibility.

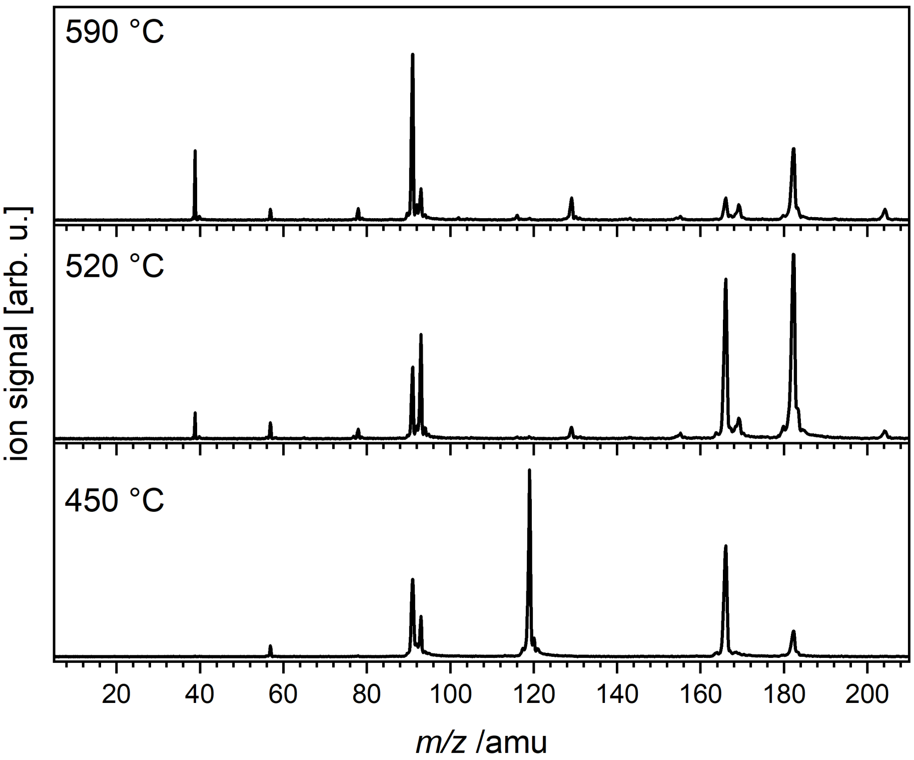

Figure S6: Pyrolysis mass spectra of the co-flow experiment at $h\nu = 9.50\,\mathrm{eV}$. Propargyl iodide at m/z 166 starts decomposing into propargyl radicals m/z 39 between 450 and 520 °C.

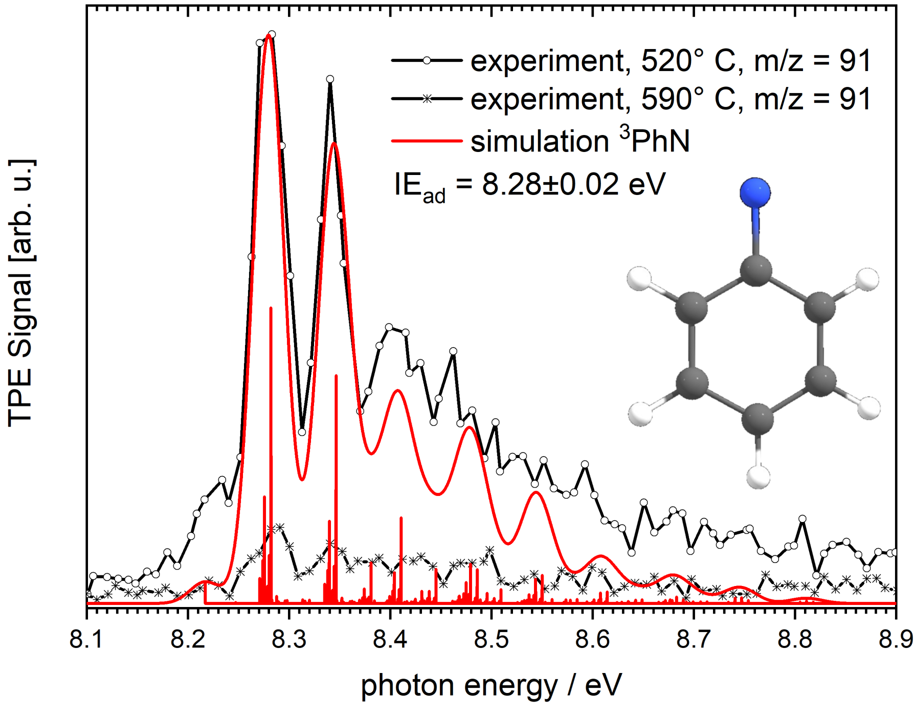

Figure S7: ms-TPES of mass channel 91 in the co-flow experiment and simulation of phenylnitrene.

S6 Potential secondary chemistry

To investigate the potential interference from secondary chemistry, we calculated the initial steps from the cyano cyclopentadienes (Cp–CN) and aniline with propargyl. In all, we find considerable barriers that hamper any reaction of propargyl even under high temperatures. Furthermore, for all Cp–CN isomers, the initial reaction products are endothermic, rendering their production unlikely. For aniline any association at the amino group is highly endothermic as well and exothermic products can only be formed at the *ortho*- or *para*-position.

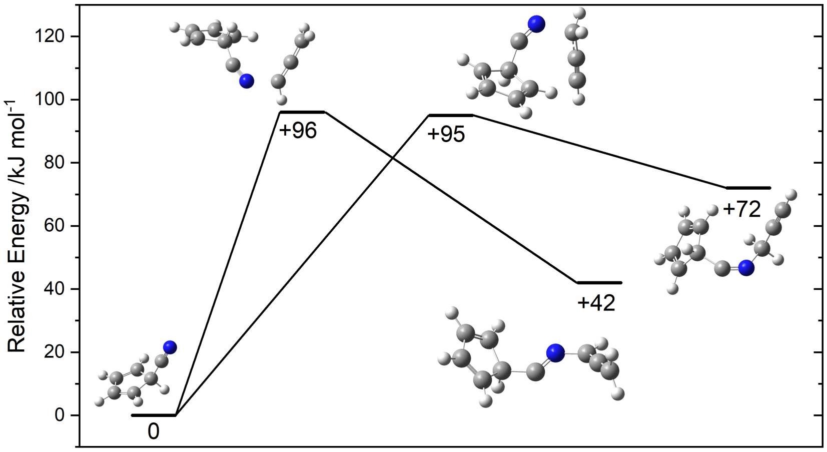

Figure S8: Potential energy surface for the reaction of 1-cyano cyclopentadiene with propargyl.

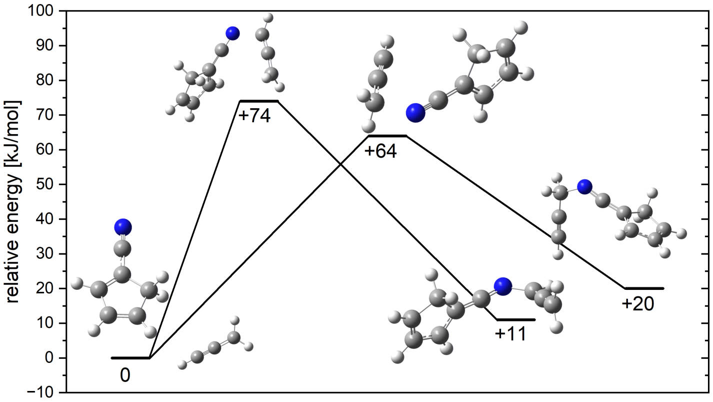

Figure S9: Potential energy surface for the reaction of 2-cyano cyclopentadiene with propargyl.

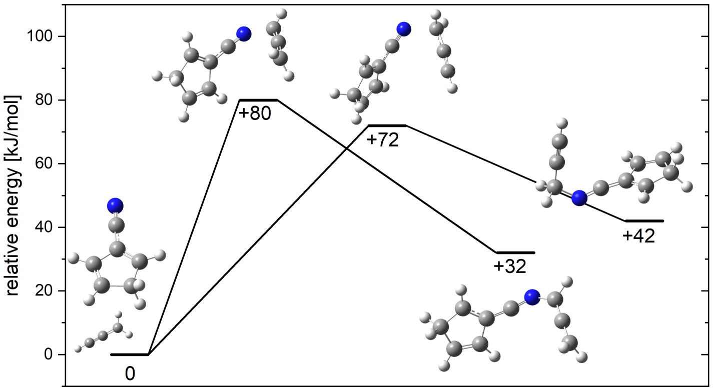

Figure S10: Potential energy surface for the reaction of 3-cyano cyclopentadiene with propargyl.

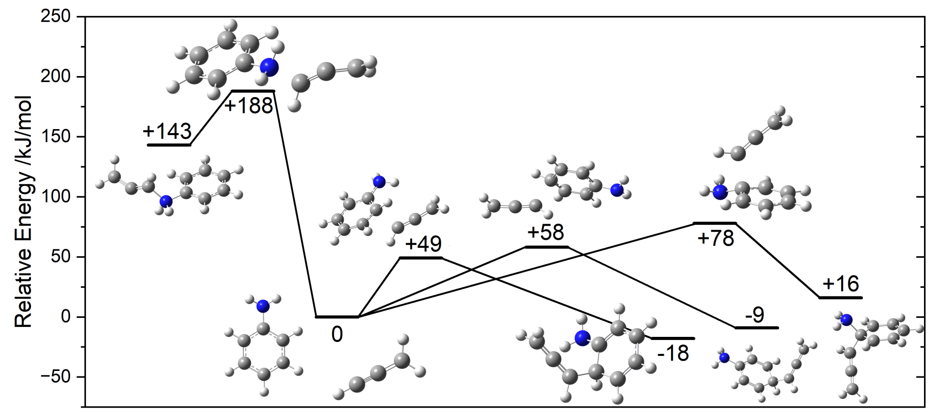

Figure S11: Potential energy surface for the reaction of aniline with the CH-site of propargyl.

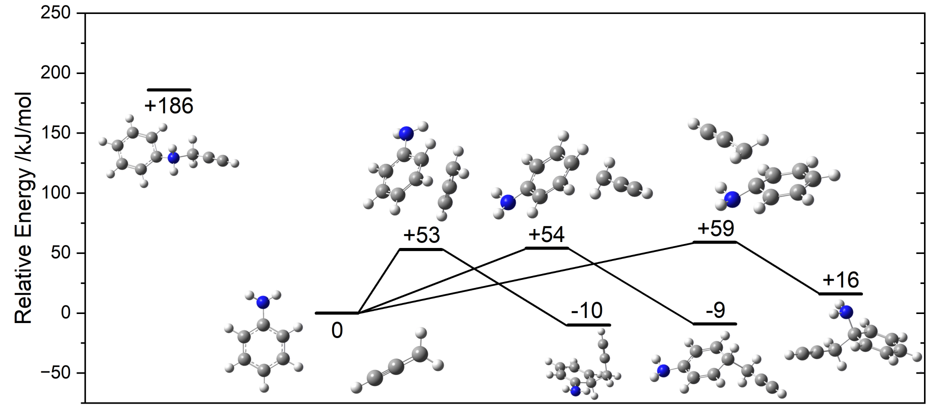

Figure S12: Potential energy surface for the reaction of aniline with the CH_2 site of propargyl.

S7 Isomers of C_9H_7N (m/z 129)

Table S1: Isomers of C_9H_7N (m/z 129), their adiabatic ionization energies (IE_{ad}) and computed relative energies to the separated reactants PhN and propargyl. Experimental values are given whenever available. Calculated values are based in CBS-QB3 computations.

Label	Molecule	IE_{ad} /eV	Rel. Energy /kJ/mol
P1	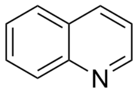	8.61±0.02 [16]	-347
P2	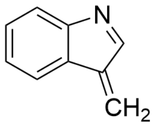	8.57	-263
P3	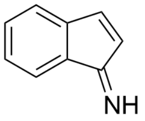	8.48	-272
P4E	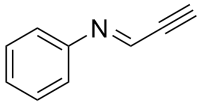	8.19	-103
P4Z	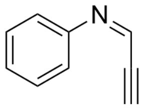	8.14	-100
P5	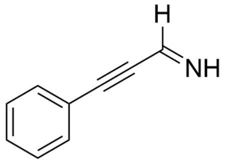	8.65	-130
P6	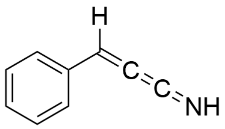	8.11	-114

Label	Molecule	IE$_{ad,calc}$ /eV
P7	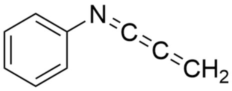	7.72
P8	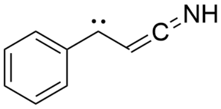	6.54
P9	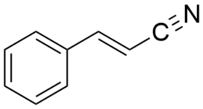	9.00 (exp)[17]
P10	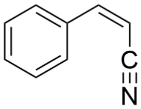	8.92 (exp)[17]
P11	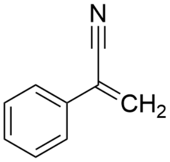	9.12 (exp)[17]
P12	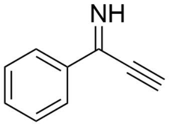	9.14
P13	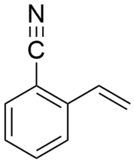	9.06 (exp)[17]
P14	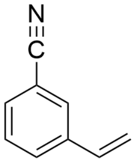	9.03

Label	Molecule	$IE_{ad,calc}$ /eV
P15	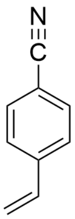	8.94
P16	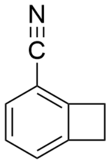	9.29
P17	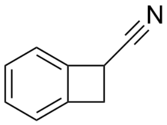	9.33

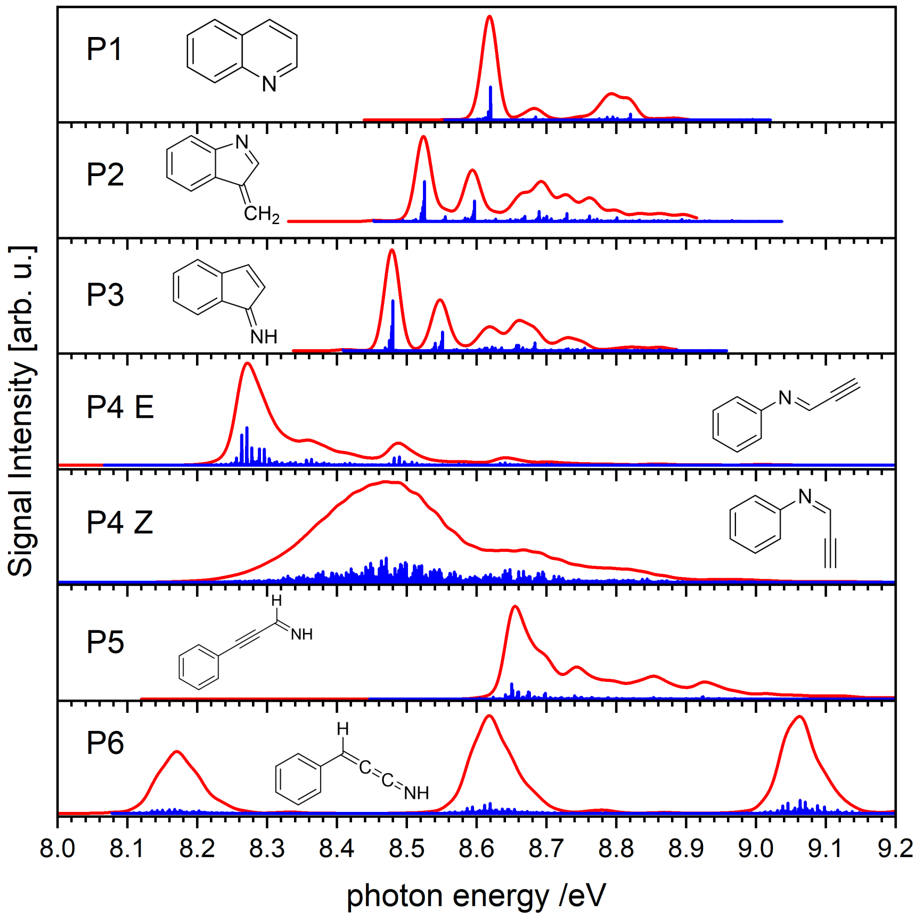

Figure S13: Franck–Condon simulations of the photoelectron spectra of possible products **P1-P6**.

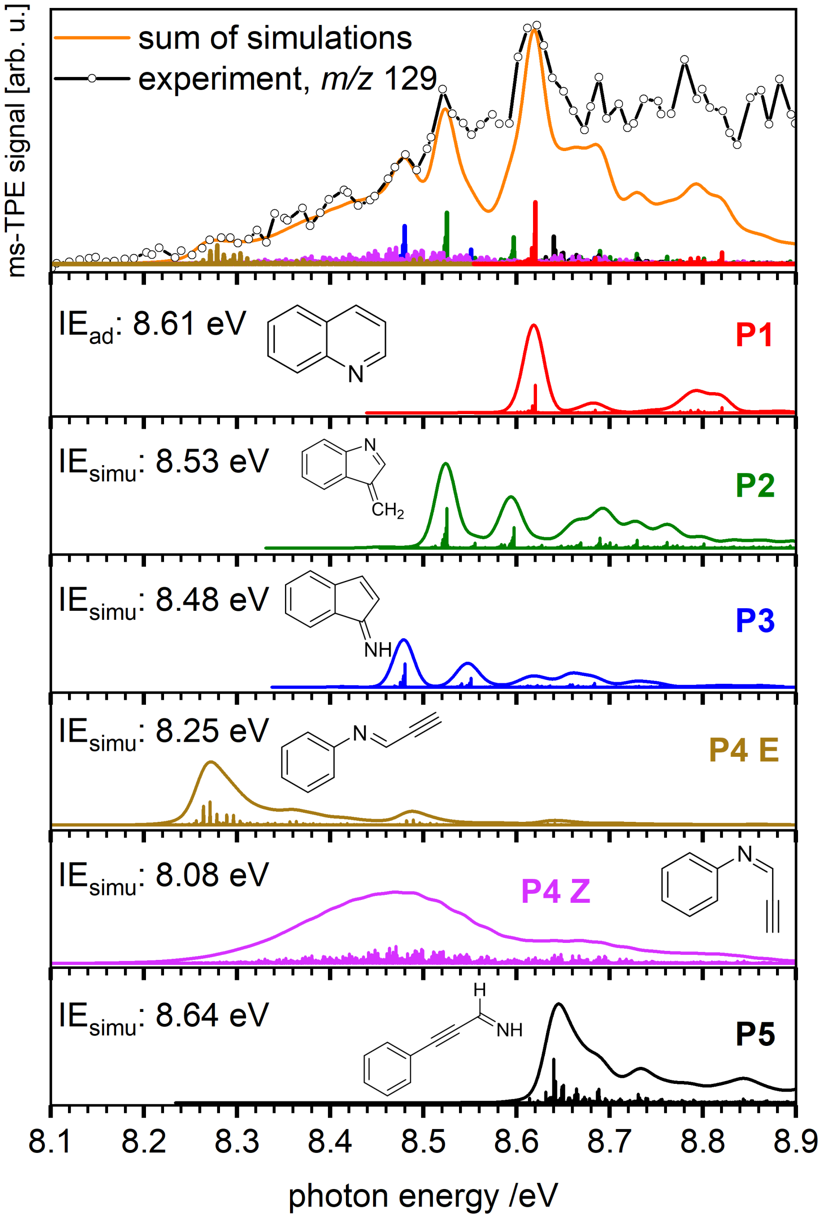

Figure S14: Experimental trace of m/z 129 in the co-flow pyrolysis experiment of phenyl azide and propargyl iodide at 520 °C. All six potential isomers were considered for the FC-simulation. The formation of P3 and P5 requires cleaving the initial C-N bond making them unlikely to be formed in the PhN + propargyl reaction. A detailed discussion is found in section S8.1.3.

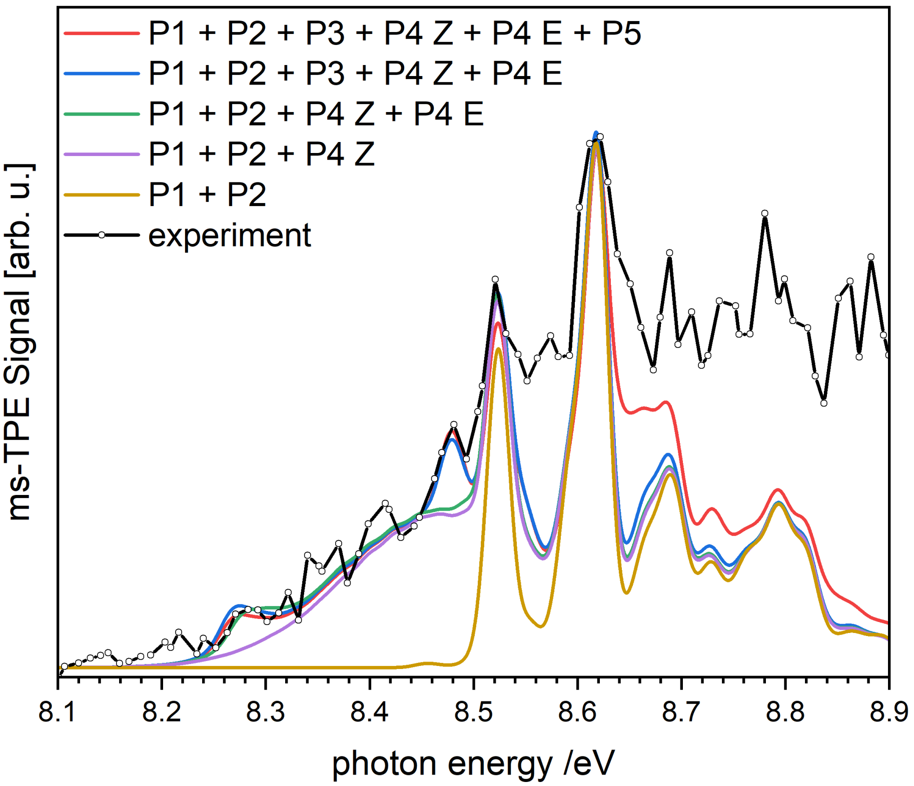

Figure S15: Overlayed Franck–Condon simulations of different sets of potential products. The spectrum can be described well using at least three contributors, **P1**, **P2**, and **P4Z**.

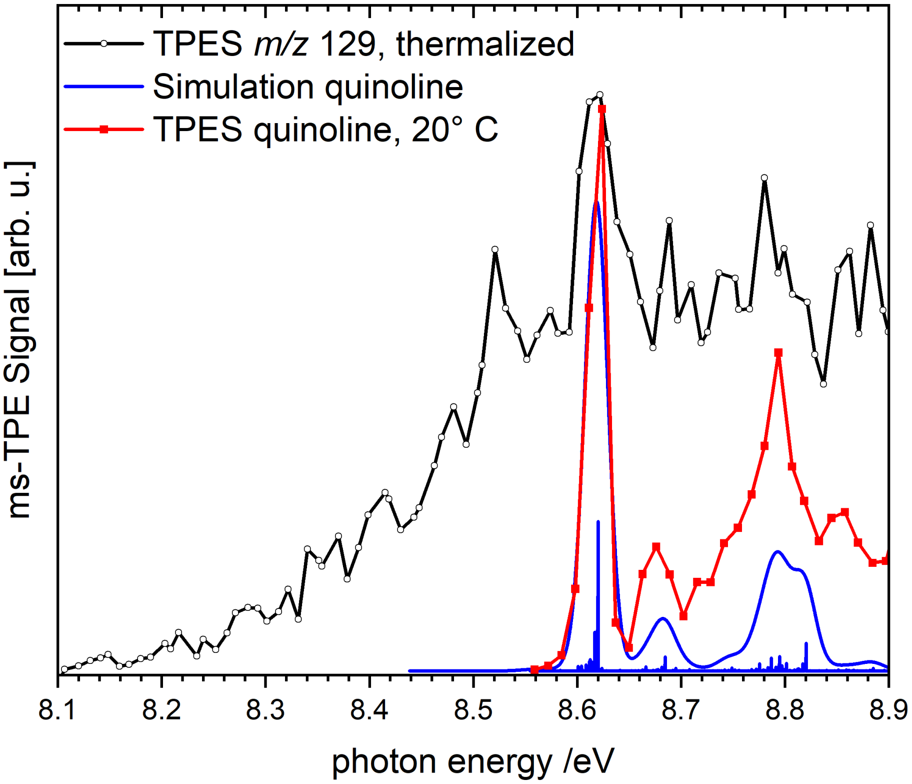

Figure S16: Experimental ms-TPES of PhN + propargyl constructed using the thermalized ion signal (black), ms-TPES quinoline at room temperature (red) and Franck–Condon simulation of quinoline using the CBS-QB3 method. When comparing the simulation and the reference spectrum, the origin band coincides perfectly with the IE_{ad} set to the experimentally determined value of 8.61 eV. However, the vibronic progressions differ slightly, with the reference spectrum showing more intense resonances than the simulated spectrum.

S8 Potential Energy Surface Calculations

The detailed description of the potential energy surface helps understand the driving forces determining the product distribution. As in the main article, we start with the description of the CH reaction and then address the CH_2 reaction. E1 and E2 refer to the isolated reactants phenylnitrene and the propargyl radical. Intermediates are abbreviated by the letter "I" and transition states are referred to as "TS".

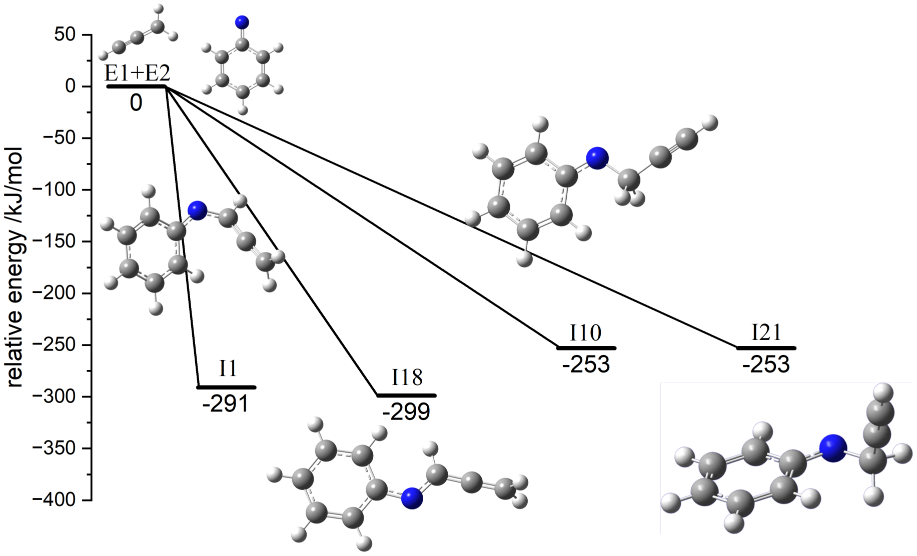

Figure S17: Possible first intermediates in the reaction between proparyl radicals and phenylnitrene. The initially formed intermediates can be formed in E– and Z–configuration. The respective E/Z–isomers have almost identical energies, however for ring closure reactions the E–isomers must first isomerize to the Z–isomers.

S8.1 CH Reactions

S8.1.1 Formation of P1 and P2

The lowest energy pathway, displayed in Fig. S18, leads over a transition state at $-219\,\mathrm{kJ\,mol^{-1}}$, resulting in a barrier of $+72\,\mathrm{kJ\,mol^{-1}}$ with respect to **I1** and forms a five-membered ring containing a terminal methylene (CH_2) group. The PES splits up right after ring closure (**I2**, see Fig. S19), into a pathway for **P1** and one for **P2**. The barriers for **P1** formation are lower and the CH_2–group can insert into the five-membered ring through a mechanism that is akin to the fulvene–benzene isomerization.[18]

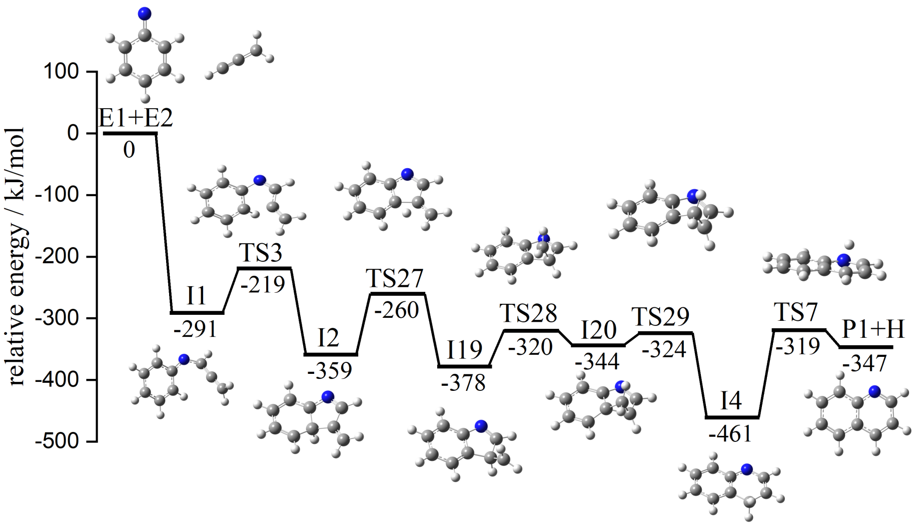

Figure S18: Lowest found Pathway via **I1** towards quinoline (**P1**).

A very similar pathway leads to the formation of 3-methylene-3H-indole (**P2**), via ring closure of the middle carbon atom. The transition state for the ring closure is the rate-limiting step with a relative energy of $-219\,\mathrm{kJ\,mol^{-1}}$ ($+72\,\mathrm{kJ\,mol^{-1}}$ relative to **I1**). Afterwards, the hydrogen atom is eliminated from the bridging carbon atom resulting in the formation of **P2**. The elimination of the hydrogen atom from **I2** is $25\,\mathrm{kJ\,mol^{-1}}$ higher than its rearrangement to quinoline (**P1**)

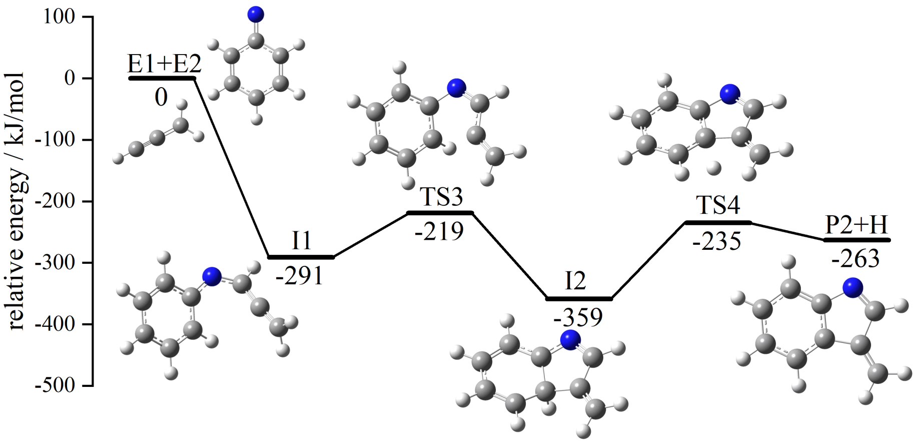

Figure S19: Pathway leading to the formation of 3-methylene-3H-indole (**P2**).

The second lowest pathway to quinoline **P1** displayed in Fig. S20 has a rate-limiting transition state at $-119\,\mathrm{kJ\,mol^{-1}}$, resulting in a barrier of $+172\,\mathrm{kJ\,mol^{-1}}$, relative to **I1**. After ring closure from the terminal CH_2 group, the bridging carbon atom transfers its hydrogen atom to the bare carbon atom in the second ring. This step leads to a partial rearomatization of the first benzene ring, thereby lowering the overall energy of the system drastically. Subsequent elimination of the excess hydrogen atom leads directly to quinoline **P1**.

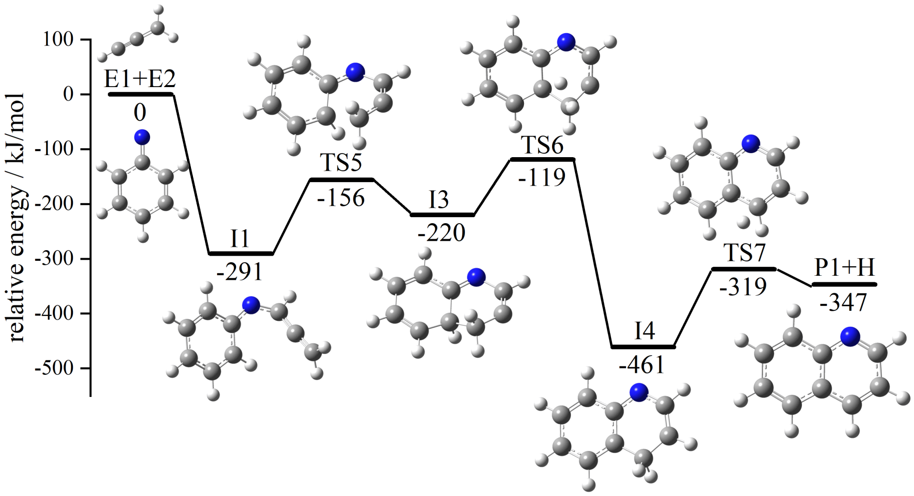

Figure S20: Secondary pathway leading to the formation of quinoline (**P1**).

Instead of the previous [1,3]H-shift in Fig. S20, a [1,2]H-shift from the CH_2 unit to the adjacent carbon atom is plausible, leading to the formation of intermediate **I5**. This pathway has a higher activation energy at $+156\,\mathrm{kJ\,mol^{-1}}$ with the rate limiting transition state at $-64\,\mathrm{kJ\,mol^{-1}}$, probably due to the inability to completely rearomatize either of the two rings.

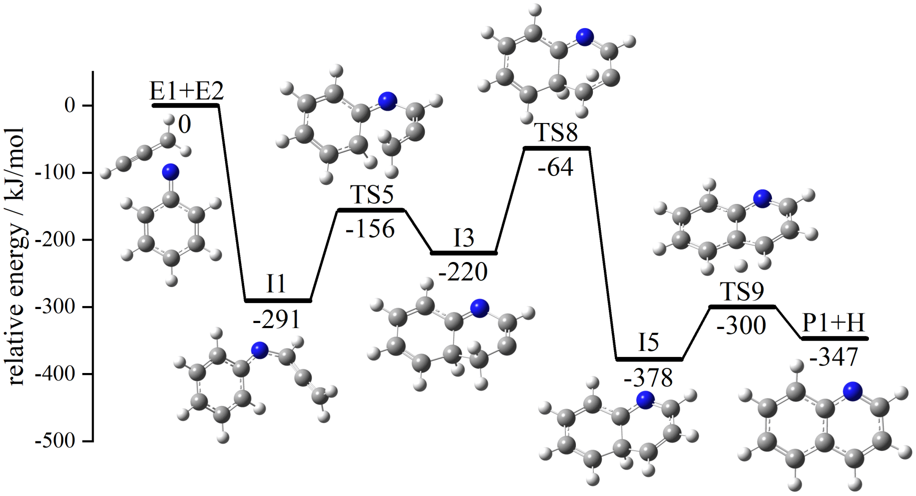

Figure S21: Reaction pathway to form quinoline (**P1**) via **I5**.

Another possibility is a hydrogen shift before ring closure, illustrated in Fig. S22. Here the hydrogen atom of the terminal CH_2 group shifts to the adjacent bare carbon atom, facilitating the subsequent ring closure. However, the hydrogen shift is energetically unfavorable with a transition state of $-62\,\mathrm{kJ\,mol^{-1}}$, hence this pathway is not competitive.

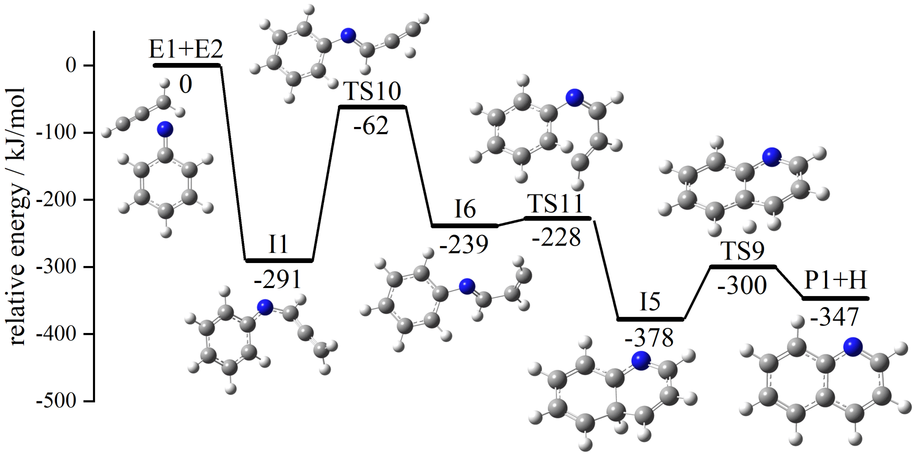

Figure S22: Alternative pathway leading to quinoline (**P1**) after a rearrangement of the open chain, via **I6**.

S8.1.2 Formation of P4E, P4Z and P7

In addition to the condensed ring products, hydrogen atom elimination could take place before the ring closure, leading to ring-open isomers. Three different products are possible, depending on the the position of the eliminated hydrogen atom. Our calculations show that only the elimination of hydrogen atoms from the former propargyl subunit in **I1** can lead to stable products, precluding the elimination of hydrogen atoms from the phenyl ring. All direct hydrogen elimination pathways are depicted in Fig. S23, where barriers of +209, +204, and $+234\,\mathrm{kJ\,mol^{-1}}$ are found.

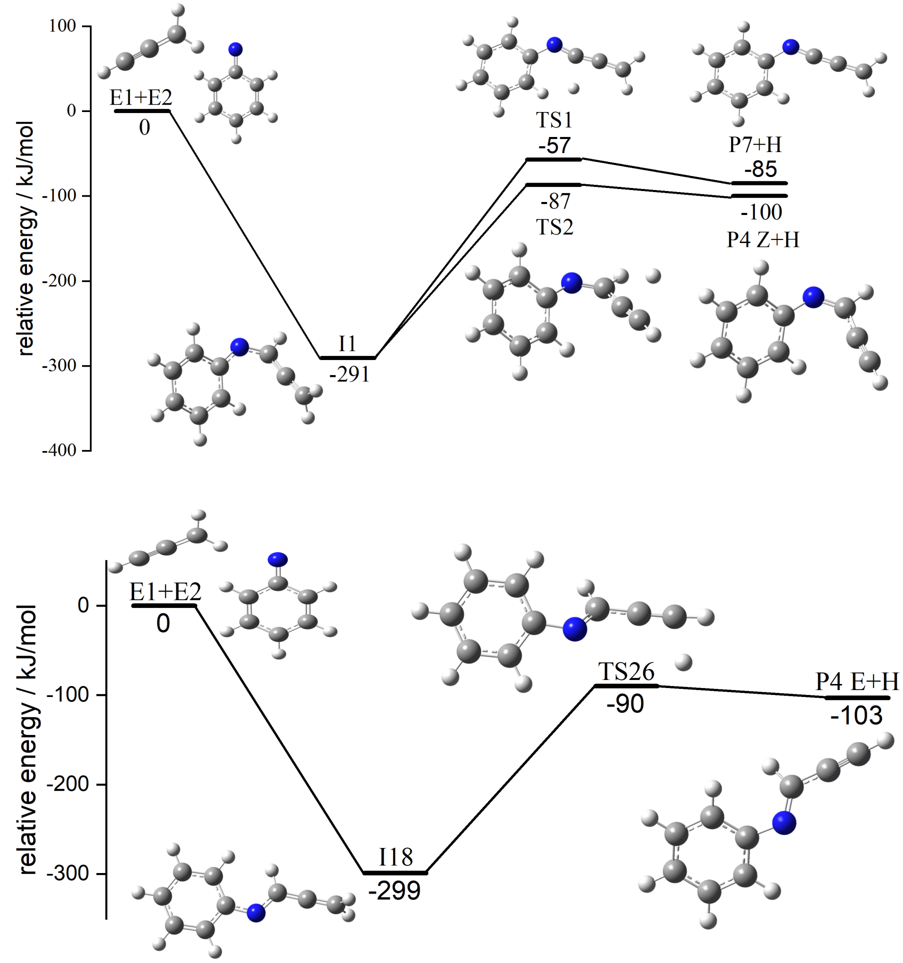

Figure S23: Pathways with direct H-elimination after recombination of the reactants, leading to three possible open-chain products **P7**, **P4 Z**,**P4 E**.

S8.1.3 On the presence of P3 and P5

The pathway in Fig. S21 also offers the possibility to another potential product **P3** (see Fig. S24). The elimination of a hydrogen from **Int5** has a barrier of $+78\,\mathrm{kJ\,mol^{-1}}$ (TS9), while the ring-contraction to **P3** has a barrier of $+213\,\mathrm{kJ\,mol^{-1}}$ (TS12) relative to **I5** and is thus unlikely to occur.

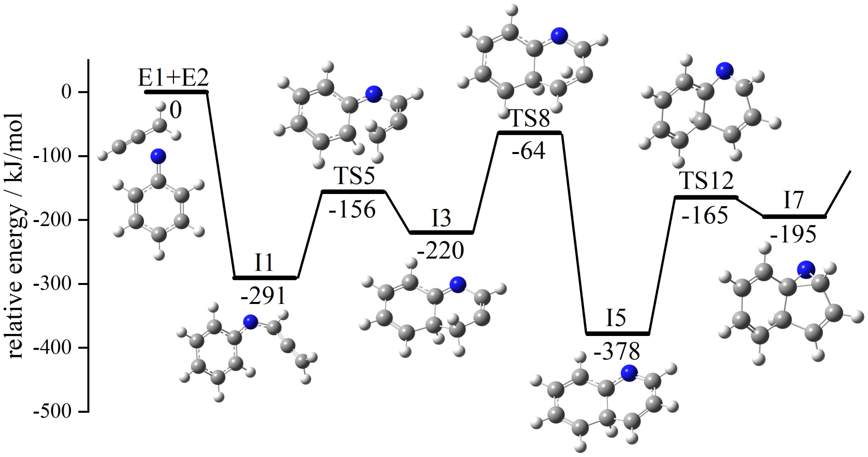

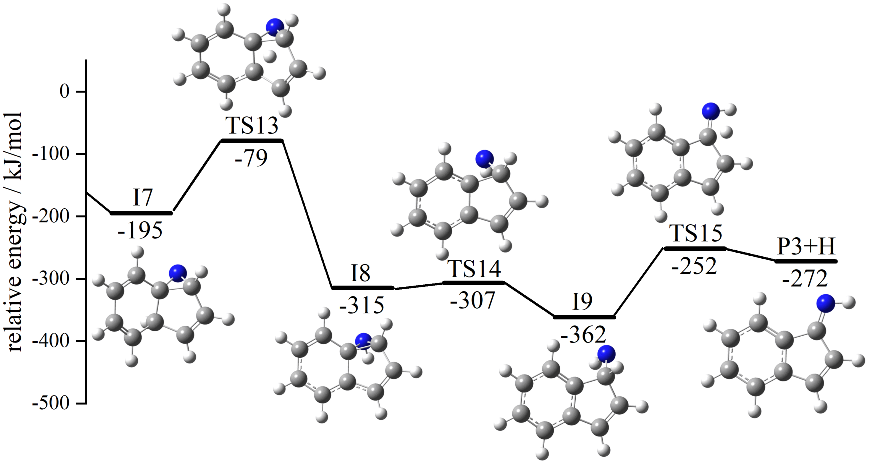

Figure S24: Pathway via **I5** to **P3**. The energy barrier towards the five-membered ring (**TS12**) is significantly higher than the H-loss (**TS9** in Fig. S21), but still lower than the separated reactants.

In this context it is advantageous to discuss the possibility of **P3** and **P5** as reaction products of the PhN + propargyl reaction. Based on their IE_{ad} and their FC-simulations, illustrated in Fig. S13 - S15, they can be considered as potential products. However, there are arguments that speak against their formation in the title reaction.

First, the structure of **P3** and **P5** require the nitrogen to be detached from the benzene ring, a process that requires substantial isomerization in subsequent intermediates. As a result, before the C_{Ph}–N scission toward the phenyl ring can occur, a new C_{Ph}–C bond has to be formed in a bicyclic intermediate with a weak C_{Ph}–N bond. These intermediates can be **I4** or **I5**, but these will yield **P1** instead.

Second, the energy of **P5** is $-130\,\mathrm{kJ/mol}$ relative to the isolated reactants. If a pathway exists that would form **P5** without any higher rate-limiting barriers, it would still need to compete with a transition state at $-219\,\mathrm{kJ/mol}$ for **P1** and **P2** in the CH-reaction. Even if a route to **P5** should exist in the CH_2-reaction, it would not affect our main finding, the formation of **P1** and **P2** via the CH-reaction.

Last, **P3** and **P5** could be formed by a secondary process, such as hydrogen-assisted secondary chemistry in the microreactor. In such an event, they would be present and contribute to the ms-TPES, but their presence would not be related to the title reaction or the formation of **P1** as a result of the CH-reaction.

When considering these arguments, the formation of **P1** is unaffected by the presence or absence of **P3** and **P5**. At the same time and despite finding no competitive pathways to either species in our PES exploration, we cannot rule out their presence.

S8.2 CH_2 Reactions

Similar to the CH reaction, the formation of a five-membered intermediate (**I17**) is possible. However, as Fig. S25 and S26 show, any further reactions toward either saturating the terminal CH group or cyclization are precluded by high barriers that are either only slightly below the reactant energies ($-21\,\mathrm{kJ\,mol^{-1}}$) or even slightly above it (+17, +18, +54, and $+56\,\mathrm{kJ\,mol^{-1}}$). We therefore conclude that these pathways do not play a decisive role in the CH_2 reaction.

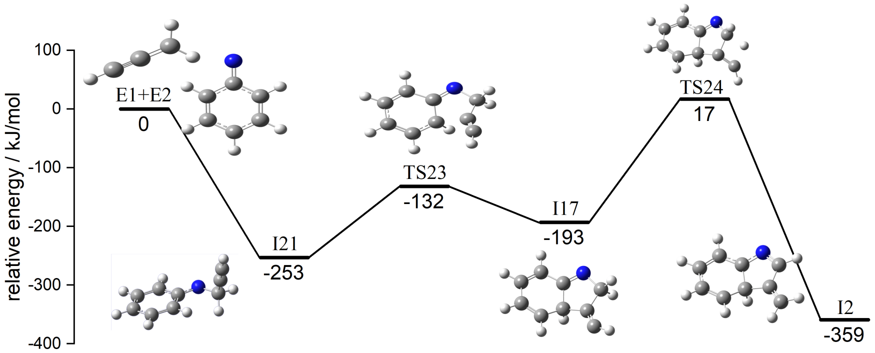

Figure S25: Pathway from **I21** to **I2**. The barrier of $17\,\mathrm{kJ\,mol^{-1}}$ precludes the formation of **P2**.

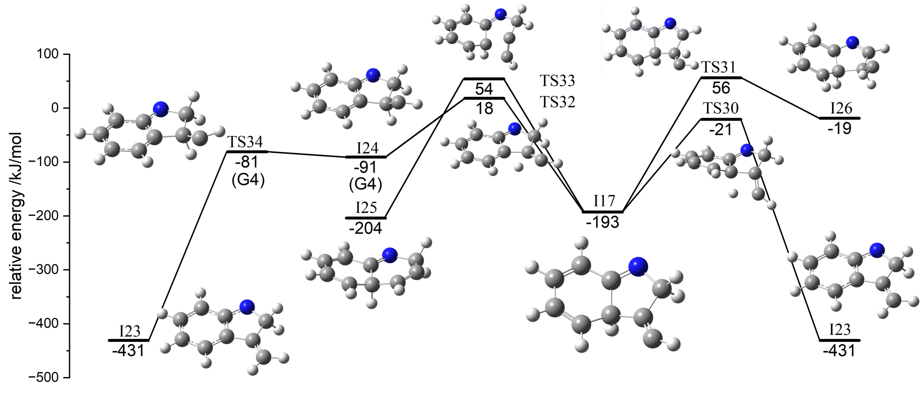

Figure S26: Possible isomerization pathways form the initially formed five-membered intermediate **I17** in the CH_2 reaction. All barriers have significantly higher barriers than the comparable pathway in the CH reaction (see Fig. S18). Computations based on G4 level of theory are noted.

The only viable pathways are the direct elimination of a hydrogen atom from the E/Z isomers **I10** and **I21**. Our computations predict transition states at −83 and $-80\,\mathrm{kJ\,mol^{-1}}$ for the elimination from **I10** and **I21**, respectively. These are the lowest barriers for the CH_2 reaction.

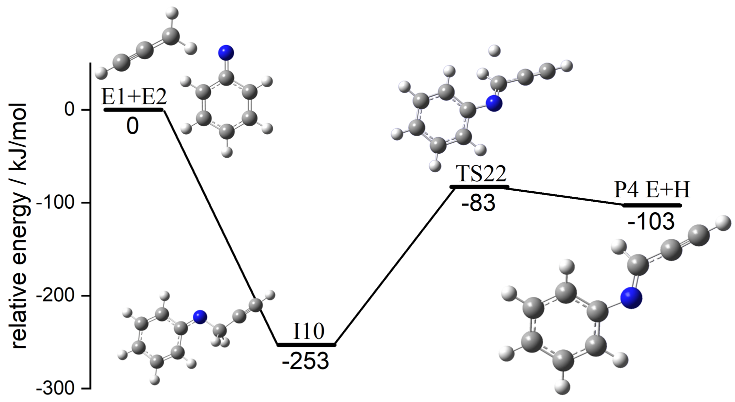

Figure S27: Pathway to **P4 E** from **I10**.

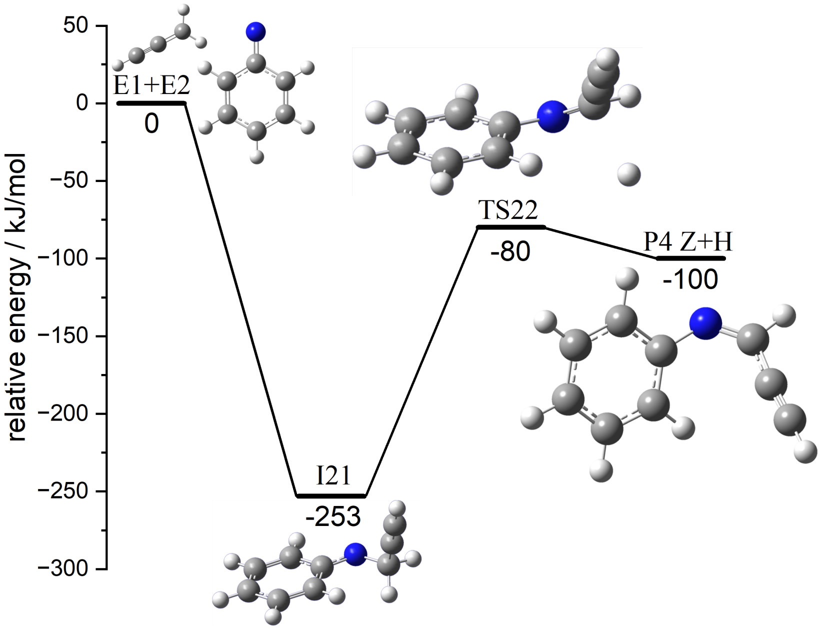

Figure S28: Pathway to **P4 Z** from **I21**.

Alternatively, a direct cyclization could occur, illustrated in Fig. S29. Although the formation of the six-membered ring is possible, the required hydrogen shift from the CH_2-group of **I11** has a transition state at $-59\,kJ\,mol^{-1}$. The second possibility, a hydrogen shift from the bridging CH has an even more energetic transition state at $+40\,kJ\,mol^{-1}$ (Fig. S30).

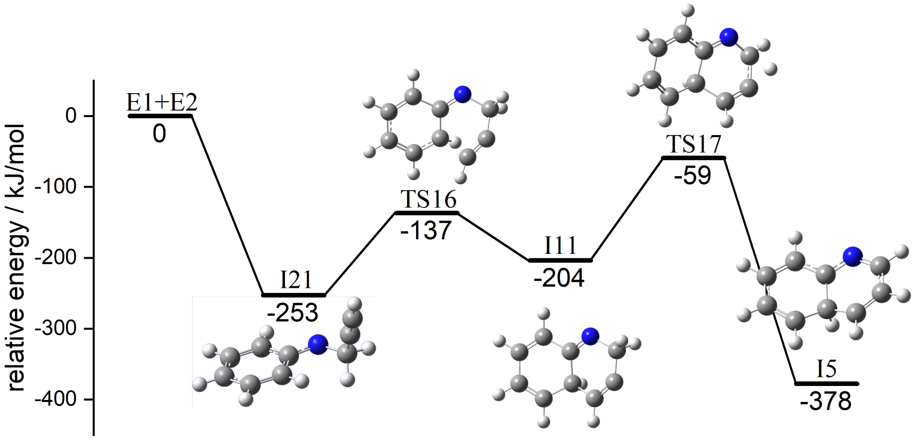

Figure S29: Pathway via **I21** to **I5**. The latter can react further to quinoline (Fig. S21).

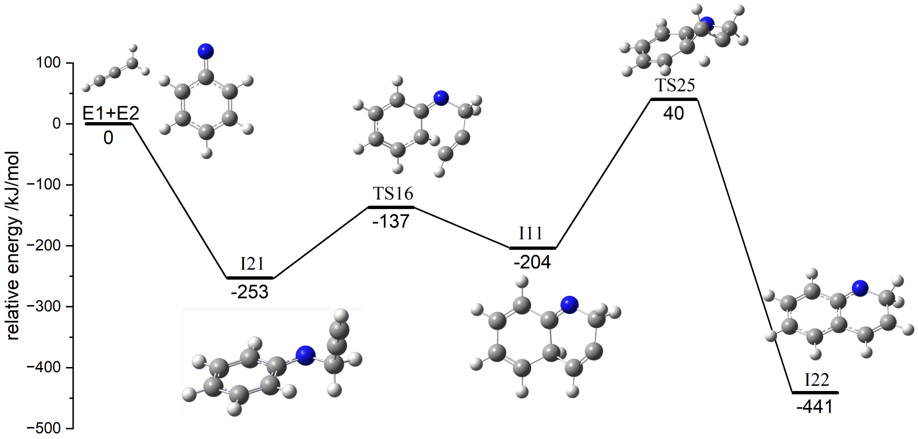

Figure S30: Pathway via **I21** to **I22**. The final transition state is above the reactant energies and is not competitive even with respect to back dissociation.

References

[1] M. Johnson, A. Bodi, L. Schulz, T. Gerber, *Nucl. Instrum. Methods Phys. Res., Sect. A* **2009**, *610*, 597.

[2] A. Bodi, P. Hemberger, T. Gerber, B. Sztáray, *Rev. Sci. Instr.* **2012**, *83*, 083105.

[3] B. Sztáray, K. Voronova, K. G. Torma, K. J. Covert, A. Bodi, P. Hemberger, T. Gerber, D. L. Osborn, *J. Chem. Phys.* **2017**, *147*.

[4] W. Tang, R. S. Tranter, K. Brezinsky, *J. Phys. Chem. A* **2005**, *109*, 6056.

[5] M. I. Mangione, R. A. Spanevello, M. B. Anzardi, *RSC Adv.* **2017**, *7*, 47681.

[6] B. Sztáray, T. Baer, *Rev. Sci. Instr.* **2003**, *74*, 3763.

[7] P. Hemberger, X. Wu, Z. Pan, A. Bodi, *J. Phys. Chem. A* **2022**, *126*, 2196.

[8] C. Fernholz, A. Bodi, P. Hemberger, *J. Phys. Chem. A* **2022**, *126*, 9022.

[9] K. P. Somers, J. M. Simmie, *J. Phys. Chem. A* **2015**, *119*, 8922.

[10] S. Gozem, A. I. Krylov, *Wiley Interdisciplinary Reviews: Computational Molecular Science* **2022**, *12*, e1546.

[11] P. Constantinidis, F. Hirsch, I. Fischer, A. Dey, A. Rijs, *J. Phys. Chem. A* **2017**, *121*, 181.

[12] M. H. Palmer, T. Ridley, S. V. Hoffmann, N. C. Jones, M. Coreno, M. de Simone, C. Grazioli, M. Biczysko, A. Baiardi, *J. Chem. Phys.* **2015**, *142*, 134301.

[13] E. Mendez-Vega, W. Sander, P. Hemberger, *J. Phys. Chem. A* **2020**, *124*, 3836.

[14] N. P. Gritsan, T. Yuzawa, M. S. Platz, *J. Am. Chem. Soc.* **1997**, *119*, 5059.

[15] J. Soto, J. C. Otero, *J. Phys. Chem. A* **2019**, *123*, 9053.

[16] J. Bouwman, B. Sztáray, J. Oomens, P. Hemberger, A. Bodi, *J. Phys. Chem. A* **2015**, *119*, 1127.

[17] J. Bouwman, A. Bodi, P. Hemberger, *Phys. Chem. Chem. Phys.* **2018**, *20*, 29910.

[18] J. A. Miller, S. J. Klippenstein, *J. Phys. Chem. A* **2003**, *107*, 7783.
